# Black Chokeberry (*Aronia melanocarpa*) Juice Supplementation Improves Oxidative Stress and Aging Markers in Testis of Aged Rats

**DOI:** 10.3390/cimb46050270

**Published:** 2024-05-08

**Authors:** Elena Daskalova, Mina Pencheva, Petko Denev

**Affiliations:** 1 Department of Anatomy, Histology and Embryology, Medical Faculty, Medical University-Plovdiv, 4000 Plovdiv, Bulgaria; elena.daskalova@mu-plovdiv.bg; 2 Department of Medical Physics and Biophysics, Faculty of Pharmacy, Medical University-Plovdiv, 4000 Plovdiv, Bulgaria; mina.pencheva@mu-plovdiv.bg; 3 Laboratory of Biologically Active Substances, Institute of Organic Chemistry with Centre of Phytochemistry, Bulgarian Academy of Sciences, 139 Ruski Blvd., 4000 Plovdiv, Bulgaria

**Keywords:** aging testis, oxidative stress, antioxidant activity, black chokeberry (*Aronia melanocarpa*)

## Abstract

Spermatogenesis is a process that continues until the end of an individual’s life, although with reduced activity with advancing age. Inflammation, oxidation, and apoptosis are events considered as predictors of pathogenesis and the development of age-related diseases observed in aged testes. The use of natural compounds with antioxidant and anti-inflammatory properties has a beneficial effect on the inflammatory and oxidative status of the aged testis. The aim of this study was to determine the effect of supplementation with antioxidant-rich black chokeberry (*Aronia melanocarpa*) juice on several markers of oxidative stress and aging in rat testis. In total, 24 male Wistar rats were divided into three experimental groups: young controls aged 2 months, old controls aged 27 months, and 27-month-old rats supplemented with black chokeberry juice at a dose of 10 mL/kg for 3 months. *A. melanocarpa* juice supplementation led to reduced oxidative stress, manifested by increased immunoexpression of nNOS, eNOS, and MAS1 in the seminiferous tubules and in the Leydig cells. The morphometrically determined tubule structure data showed no significant differences between the three groups. However, the intensity of the immunoreaction for TRK-C and NT3 in Leydig cells was demonstrably higher in the supplemented old animals compared with the old controls. There was a significantly higher number of blood vessels around the seminiferous tubules in the supplemented animals compared to the old controls. These data indicate that supplementation with *A. melanocarpa* juice slows down aging processes in the testis and preserves the functional activity of Leydig cells.

## 1. Introduction

Aging is a natural process involving irreversible changes in all organs due to a number of endogenous and environmental factors, and testicular changes are one of the effects of aging on the male reproductive system. These changes can lead to a reduction in sperm quality and quantity, which is also associated with a reduction in testosterone levels [1,2,3]. Furthermore, age-related decline in testicular function also affects overall health status and life quality [4]. There is no definite time for the onset of progressive age-related testicular involution [5]. Age-related changes in the testis affect both the spermatogenic epithelium and the Sertoli and Leydig cells, in which degenerative manifestations are observed [6]. The most common histological finding in aging testes is the variation in spermatogenesis in the seminiferous tubules. In both humans and rodents, age-related atrophy begins focally, with atrophic tubules in the testis adjacent to those with normal spermatogenesis. In testicular tissue with its characteristic high levels of metabolic activity and cell replication, oxidative stress can be particularly damaging, a fact that makes antioxidant capacity particularly important [5,7].

According to the findings of different studies, sperms are more susceptible to oxidative stress than other cells because of the limited amount of cytoplasm in mature sperm, the concentration of ROS-suppressing antioxidants in sperm, and the high levels of unsaturated fatty acids in the sperm structure [8]. 

It has been proven that in rats, the expression of enzymatic and non-enzymatic antioxidants decreases with age, leading to increased damage from oxidative stress. In addition, the levels of glutathione, an antioxidant, decrease in older rats [9]. 

Low levels of reactive oxygen species (ROS) are required for normal sperm function (e.g., capacitation, hyperactivation, acrosomal reaction, and fertilization) [10]. Recently, it has been shown that elevated levels of oxidative stress in elderly mice reduce testicular steroidogenesis and impaired glucose metabolism is correlated with adiponectin receptor reduction [11]. Since the presence of ROS and the resulting oxidative stress are closely related to apoptosis, the increase in apoptotic events described in human testis during aging can be explained, at least in part, by the accumulation of ROS in aged testis [12].

In recent years, the function of the renin–angiotensin system (RAS) as an endocrine regulator of renal and cardiovascular function has undergone revision [13]. Increasing importance is being given to local RAS in different organs. The identification of several components of the RAS, such as prorenin, renin, angiotensinogen, angiotensin (Ang) I, Ang II, Ang-converting enzyme (ACE), and ACE2, in human and mammalian testis and epididymis strongly support the presence and involvement of a local RAS in male reproduction [14,15,16,17,18]. The expression of elements of this local RAS has been described in different parts of the human reproductive tract [13,19]. A large number of genetic and protein analyses confirmed the presence of RAS components in the testes, seminal vesicles, epididymis, and prostate in humans and animals [14,20]. ACE2 is a human homolog of ACE and is highly expressed in the heart, kidneys, and testes of humans and animals. Several different studies found that ACE2 expression in the testes is limited to Leydig cells in rats and Leydig and Sertoli cells in humans [21,22,23]. The authors also suggest the role of ACE2 in controlling testicular function and possibly regulating steroidogenesis or other functions in Leydig cells [24]. 

The function of neurotrophins is to maintain the survival and differentiation of neurons, but it has also been demonstrated that all known neurotrophins and their receptors are expressed in the testes [25]. In animals, three members of the tyrosine protein kinase receptor (TRK) family are known: TRK-A, which binds to the nerve growth factor (NGF); TRK-B, which binds to the brain-derived neurotrophic factor (BDNF) and neurotrophin-4 (NT-4); and TRK-C, which binds to neurotrophin-3 (NT3) [26]. During testicular embryogenesis, neurotrophins and their receptors are expressed in germ cells throughout development and in Sertoli and Leydig somatic cells [26]. Neurotrophic factors have also been shown to play a role in the postnatal differentiation of Leydig cells and the regulation of their steroidogenic activity by autocrine and paracrine mechanisms [27].

Nitric oxide (NO) is a reactive nitrogen species and appears to be a key molecule in mediating important physiological mechanisms such as angiogenesis, growth, puberty, and aging [28]. Nitric oxide synthase (NOS) is responsible for the synthesis of nitric oxide. So far, three NOS isoforms have been discovered in animal cells, namely neuronal NOS (nNOS and NOS 1), inducible NOS (iNOS and NOS 2), and endothelial NOS (eNOS and NOS 3) [29]. In the male reproductive system, NO plays a vital role in normal reproduction and the regulation of germ–cell apoptosis [28]. In addition, this supposedly simple molecule is involved in other roles, such as the evolution of germ cells, the connections between Sertoli cells and germ cells in the blood–testis barrier, and germ–cell apoptosis. Furthermore, because of its widespread distribution in both normal and diseased testicular tissue, NO is considered a key factor in male fertility [28]. The physiological roles of NO/NOS are diverse due to the various NO-mediated signaling pathways [30,31]. For example, NO is involved as a crucial regulator in inflammation [31], and it is a physiological regulator of the endocrine system [32]. In the testes, it has been shown that NOS regulates a range of functions, including sperm motility and maturation, as well as apoptosis of germ cells [32]. Notably, the first significance of NO in sperm motility stems from localization studies demonstrating the presence of all three types of NOS (eNOS, iNOS, and nNOS) in spermatozoa [33]. These results appear to suggest the crucial role of NO/NOS in the normal functioning of spermatozoa [32].

The application of natural products aimed at improving testicular function in elderly individuals is a valuable preventive strategy. Nutrients such as polyphenolic compounds, found in plants, can stimulate testosterone production through various regulatory mechanisms [34]. The fruits of black chokeberry (*Aronia melanocarpa)* are rich in polyphenols and other antioxidants, such as vitamin C and E, and have proven anti-inflammatory and antioxidant properties, which underlie their hepatoprotective, immunomodulatory, antimutagenic, anticancer, lipid-lowering, antidiabetic, and antihypertensive effects [35]. Recently, the products and extracts from black chokeberry have attracted serious scientific interest regarding their anti-aging properties and geroprotective activities [35,36,37,38,39,40,41,42]. Our previous studies using the same model of healthy naturally aged Wistar rats demonstrate that supplementation with *A. melanocarpa* fruit juice diminishes age-related remodeling of coronary arteries, reveals neuroprotective effects, and improves cognitive and locomotor functions of aged rats [41,42]. However, the literature lacks experimental evidence on the influence of *A. melanocarpa*-based extracts and products on the aging process in testicles. Thus, the assumption that the antioxidant and anti-inflammatory properties of *A. melanocarpa* may influence age-related processes in the testis determined the aim of this study. Therefore, the aim of the study was to investigate the effect of supplementation with polyphenol-rich black chokeberry juice on some structural and functional changes in the testis in spontaneously aging old rats.

## 2. Materials and Methods

### 2.1. Aronia Melanocarpa Juice Analysis and Composition 

The same model of healthy naturally aged Wistar rats and the same juice were used in our previous study, which contains a detailed description of juice preparation and chemical analysis [41]. Briefly, five kilograms of *A. melanocarpa* frozen fruit were defrosted at room temperature and homogenized in a laboratory blender. The homogenate was transferred into a brown glass bottle and incubated in a thermostatic shaking water bath at 60 °C for one hour. After that, the pulp was filtered through a cheesecloth, and the liquid fraction was centrifuged and used for the study. Total polyphenol content was determined using the Folin–Ciocalteu reagent. HPLC analyses of polyphenols and sugars were performed on the HPLC system (Agilent 1220, Agilent Technology, Santa Clara, CA, USA, using UV-Vis and refractive index detectors, respectively [41]. The data retrieved from [41] are shown in Table 1.

### 2.2. Animals

The detailed animal protocol is given in the study by Daskalova et al. [41]. Male Wistar rats (*n* = 24) were provided by the Vivarium of Medical University, Plovdiv, where they were maintained under standard laboratory conditions (housed in polypropylene cages in a controlled clean air environment at a temperature of 22 ± 3 °C, a 12 h light/dark cycle, and relative humidity of 60 ± 5%). The rats were divided into 3 groups: (1) young controls (CY)—aged 2 months without supplementation (*n* = 8); (2) old controls (CO)—aged 27 months without supplementation (*n* = 8); and (3) (A) group—27-month-old animals, supplemented orally with *A. melanocarpa* juice (10 mL∙kg^−1^), diluted to a ratio of 1:1 in their drinking water for 105 days (*n* = 8). Rats were on a standard rodent chow (containing 13.45% protein, 51.6% carbohydrate, and 3.40% fat) and tap water ad libitum. The daily dose of juice was calculated for every animal after body weight measurement (twice a month). The animals from the A group received clear water after ingesting the daily dose of diluted juice. For the whole experimental period, every animal consumed approx. 440 mL fruit juice. At the end of the experimental period, the animals were anesthetized with i.m. Ketamin 90 mg/kg/Xilazine 10 mg/kg, weighed and measured (nasoanal length and abdominal circumference), and euthanized by cervical decapitation. After that, a bilateral orchiectomy by a median scrotal incision was performed. Each right testis was weighed (absolute weight) and the relative weight was calculated (testis weight (g)/body weight (g) × 100). Testicles were fixed in 10% neutral formalin and embedded in paraffin after fixation. Routine histological, immunohistochemical, morphometric, and statistical analyses were performed.

### 2.3. HE Staining 

Routine hematoxylin-eosin staining was performed according to standard methodology as follows: dewaxing sections; rehydrating using descending grades of alcohol to water; removing fixation pigments if necessary; staining in an alum hematoxylin for 5 min; washing well in running tap water for 5 min until sections are ‘blue’; differentiating in 1% acid alcohol (1% HCl in 70% alcohol) for 5–10 s; washing well in tap water (10–15 min); staining in 1% eosin Y for 10 min; washing in running tap water for 1–5 min.; dehydrating using alcohols, followed by clearing and mounting.

### 2.4. Immunohistochemistry

The sections (5 µm-thick) obtained from rat testicles were deparaffinized and subjected to the immunohistochemical analysis using monoclonal antibody (TRK-C, 1:1000 (sc-517245); NTR3, 1:100 (sc-376561) (Santa Cruz Biotechnology, Inc., Heidelberg, Germany)) and polyclonal antibody (nNOS1, 1:100 (E-AB-70065); eNOS3, 1:300 (E-AB-32268); MAS1, 1:200 (E-AB-67951) (Ellabscience Biotechnology Inc., Houston, TX, USA)). The immunohistochemical protocol is described in detail in our previous article (Daskalova et al.) [42]. All microphotographs were taken using a Leica DM3000 LED microscope (Leica Microsystems, Wetzlar, Germany), combined with a Flexocam C3 digital camera (Leica Microsystems, Wetzlar, Germany). 

### 2.5. Morphometric Analysis

The morphometric analysis involved serially sectioned tissue slices 5 µm in thickness, obtained from rat testicles.

The quantitative morphometric study included the following:

− Thickness of the epithelium of seminiferous tubule in μm;
− Mean perimeter of seminiferous tubules in μm
− Surface area of the seminiferous tubules in μm^2^;
− Average number of blood vessels surrounding one tubule;
− Average number of spermatogonia, spermatocytes, and spermatids in the seminiferous tubules.

The image analyzer was first automatically calibrated to convert the measurement units (pixels) generated by the image analyzer into actual units (μm) of measurement.

All measurements involved five slices per animal and an examination of all cross-sections of the seminiferous tubules present. The seminiferous tubule parameters were established by measuring all tubules with a circular cut in the slice at magnification ×100. Six different locations were measured for the thickness of the spermatogenic epithelium of each tubule. The blood vessels surrounding a duct were counted, and vessels that had already been counted were not counted for the other adjacent ducts [5,43,44]. Quantitative analysis of spermatogenesis was performed by counting the nuclei of each type of spermatogenic cell only on circularly sectioned seminiferous tubules. Five sections from each animal were counted using the Meistrich and Hess method [45]. The measurements were performed manually using the LAS X software (Leica Microsystems, Wetzlar, Germany).

### 2.6. Statistical Analysis

The results are presented with mean values and standard deviation (SD). For the comparison of three means, One-Way ANOVA with Post Hoc Multiple Comparisons using Tukey’s HSD was used. To compare the two means, the Independent Sample Test was used. For the non-parametric test, Mann–Whitney’s Two Independent Sample Test was used. The correlation between the area of tubules and the perimeter of tubules with spermatogenic epithelium thickness was measured by Pearson’s coefficient (r). *p* < 0.05 was considered significant. Statistical analysis was carried out using IBM SPSS Statistics (v25). 

## 3. Results

### 3.1. Right Testis Weight

Comparison of the right testicular weight between experimental groups revealed significant differences between the CY and CO group (*p* < 0.05) and between the A and CY group (*p* < 0.05) (Table 2), which is a manifestation of natural age-related changes.

### 3.2. Routine HE Staining

**Figure 1:**
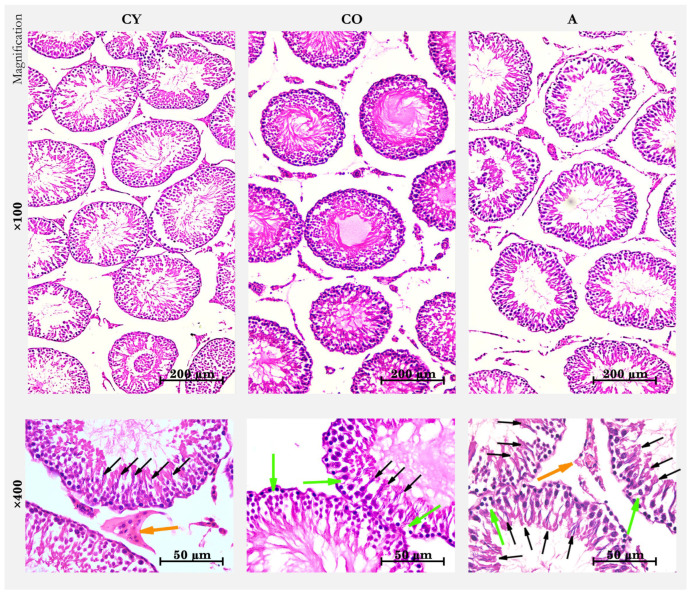
Rat testis, HE staining, magn. ×100/×400; CY—young controls, CO—old controls, and A—*A. melanocarpa* juice-supplemented group; green arrows—cell vacuolization, orange arrows—Leydig cells, and black arrows—tails of mature sperm, a sign of active spermatogenesis.

The histomorphological data are presented in Figure 1. Standard testicular HE staining in the CY group showed the testicular parenchyma of densely arranged seminiferous tubules with narrow interstitial spaces containing a scant amount of loose connective tissue. The basal lamina of the tubules presented as a fine layer. The seminiferous tubules presented with a stratified epithelium of Sertoli cells distinguished by large nuclei and spermatogenic cells including spermatogonia, spermatocytes, round early spermatozoa, and elongated late spermatozoa arranged in this order from the basal to the adluminal department of the tubule. A large percentage of tubules showed signs of active spermatogenesis with the presence of tails of mature spermatozoa in the lumen. Leydig cells were visualized in the interstitial spaces between the tubules, accompanied by quite a few blood vessels (Figure 1).

In standard HE staining testes, the CO group showed marked vacuolization of the thinned spermatogenic epithelium. The smaller amount of spermatozoa and the smaller number of tubules with signs of spermatogenesis were noticeable when compared with young controls. The basal lamina of the tubules was thickened and strongly undulating. There was obvious enlargement of the interstitial spaces. Leydig cells were without visible external changes, except for the reduced number of blood vessels accompanying them (Figure 1). In the A group, HE staining showed thickened and wavy basal lamina of the tubules, similarly to the old controls. The seminiferous tubules presented with visibly preserved Sertoli cells, a relatively greater number of tubules with active spermatogenesis, and a greater number of mature spermatozoa in the tubule lumen (Figure 1). Cells with vacuolization of the cytoplasm were also observed in the spermatogenic epithelium. The presence of a rich blood supply with abundant blood vessels adjacent to the Leydig cells was impressive (Figure 1). The tubules in the *A. melanocarpa*-supplemented group were more densely arranged, and no changes were observed in the amount and distribution of collagen fibers in the intertubular spaces. Therefore, it is very likely that this is related to the increased number of blood vessels around the tubules that we detected. 

Table 3 presents the data from the quantitative analysis of spermatogonia (Sg), spermatocytes (Sc), and spermatids (St) in the seminiferous tubules of rats from the three experimental groups. Statistical analysis of the data showed significant differences between groups on all three parameters as follows: CO vs. CY (*p* < 0.05); A vs. CO (*p* < 0.05).

### 3.3. Morphometric Analysis of Tubules Morphology

**Figure 2:**
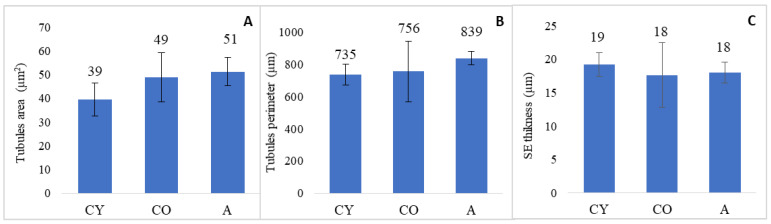
Morphometric analysis of tubules morphology. (**A**) Tubule area (µm^2^); (**B**) tubule perimeter (µm); (**C**) spermatogenic epithelium thickness (SET) (µm). Results are presented as mean value ± standard deviation. CY—young controls, CO—old controls, and A—*A. melanocarpa* juice-supplemented group.

The results of the morphometric analysis of the structure of the curved seminiferous tubules are presented in Figure 2.

**Figure 3:**
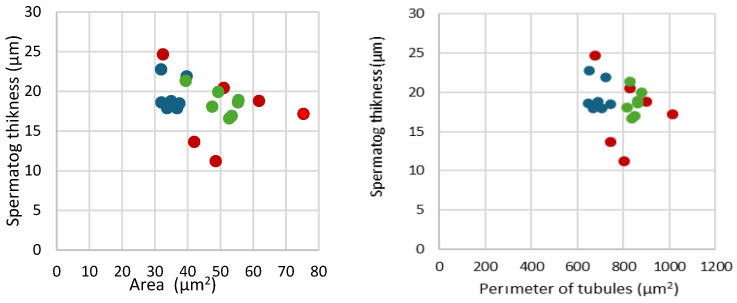
Correlation of area of tubules and perimeter of tubules with spermatogenic epithelium thickness (cases of the groups are colored as follows: CY—blue; CO—red; A—green).

Statistical analysis showed that the differences between the three experimental groups in terms of tubule area and tubule perimeter, and the thickness of the spermatogenic epithelium did not reach significance. Correlation analysis of the three tubular morphology parameters showed no significant correlations among them (Figure 3).

**Figure 4:**
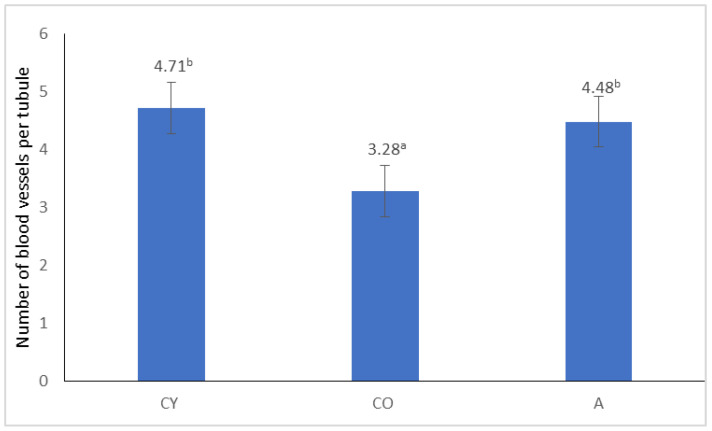
Morphometric analysis of the average number of blood vessels surrounding a tubule. Results are expressed as mean values ± standard deviation. There are no significant differences among values marked with the same superscript letters (*p* < 0.05). CY—young controls, CO—old controls, and A—*A. melanocarpa* juice-supplemented group.

Figure 4 shows the data from morphometric analysis of the mean number of blood vessels surrounding the seminiferous tubules in the three experimental groups of rats. This result conclusively confirms the finding in the histological description.

### 3.4. Immunohistochemistry

#### 3.4.1. TRK-C and NT3 Immunoreactions

**Figure 5:**
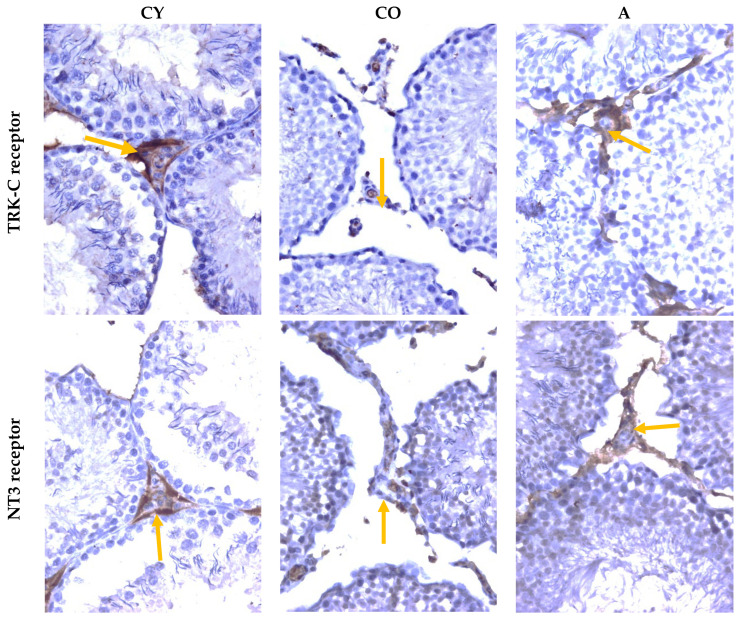
Rat testis. TRK-C immunoreaction, NT3 receptor immunoreaction, ×400; yellow arrows—Leydig cells. CY—young controls, CO—old controls, and A—*A. melanocarpa* juice-supplemented group.

Figure 5 shows micrographs of the immunoreactions for TRK-C and NT3 in rat testis.

The semiquantitative analysis of the intensity of the immune response for TRK-C and NT3 in Leydig cells showed a decrease in the CO group relative to CY, which is a manifestation of the age-related process. In *A. melanocarpa* juice-supplemented animals, a higher intensity of the immune response for TRK-C and NT3 was reported relative to CO, which is an expression of increased activity of neurotrophic factors in the Leydig cells of the testis (Table 4).

#### 3.4.2. nNOS, eNOS, and MAS1 Immunoreaction

**Figure 6:**
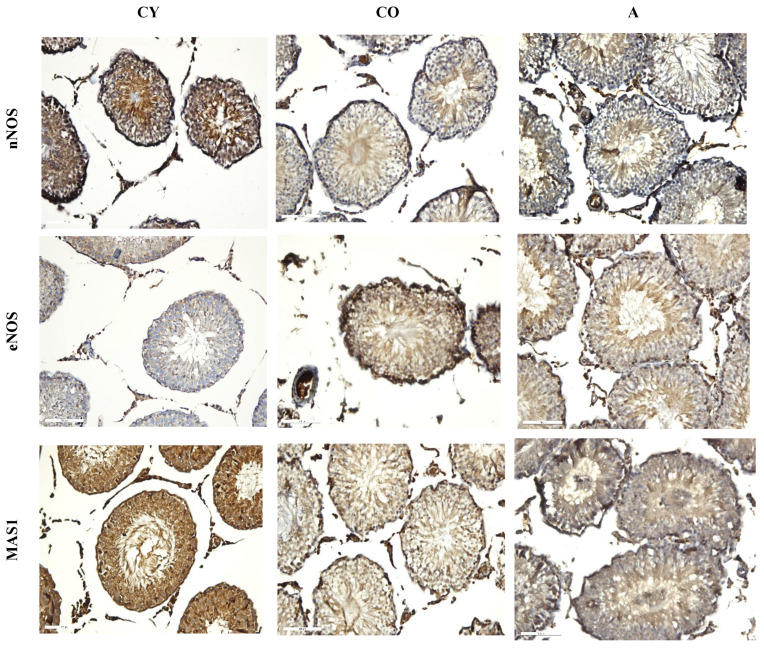
Rat testis nNOS, eNOS, and MAS1 immunoreaction, ×200. CY—young controls, CO—old controls, and A—*A. melanocarpa* juice-supplemented group.

Figure 6 shows the micrographs of the immunoreactions for nNOS, eNOS, and MAS1 in rat testis. We found that in the seminiferous tubules, nNOS in the A group presented a higher percentage in elongated spermatids and mature spermatozoa, which was less in round spermatocytes. In the CO group, nNOS is mainly visualized in mature spermatids. In the CY group, we found an increased expression of nNOS in both elongated spermatids and mature spermatozoa and in round spermatids and undifferentiated spermatogonia (Figure 6, Table 5). In the interstitium, we found that in all three groups (CY, CO, and A) nNOS was highly expressed in Leydig cells and in the endothelium of blood vessels. 

Analysis for eNOS showed higher expression levels in the seminiferous tubules of the A group relative to both the CO and CY groups. In the A group, eNOS was mainly detected in elongated spermatids and mature spermatozoa, poorly visualized in round spermatocytes. In the CO group, eNOS is diffusely presented in all seminiferous tubule cells (undifferentiated spermatogonia, round spermatids, elongated spermatids, and mature spermatozoa), but distinctly visualized in spermatogonia and myofibroblast cells. In the CY group, eNOS is poorly represented in elongated spermatids and mature spermatids. In the interstitium, eNOS presented more intense expressions in the A group and weaker expressions in the CO and CY groups, whereas, in the endothelium of blood vessels, it was not visualized in all three groups (Figure 6, Table 5).

Regarding MAS1 receptor expression in the CY group, we found a high intensity of expression in all cells in the seminiferous tubules, in Leydig cells, and in the endothelium of the blood vessels in the interstitium. In the A and CO groups, the MAS1 receptor was visualized in round, elongated spermatids and mature spermatids in both groups, with higher response intensity in the A group. In the interstitium, MAS1 was visualized predominantly in Leydig cells and was not detected in the endothelium of blood vessels in both the A and CO groups (Figure 6, Table 5).

**Figure 7:**
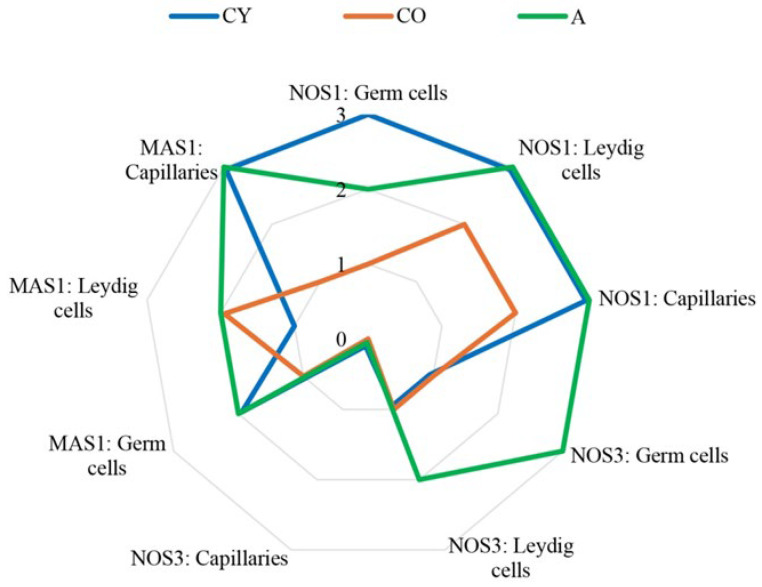
Diagram of immunoexpression intensity for nNOS, eNOS, and MAS1 in rat testis.

Figure 7 visually represents that the intensity values of immunoreactions for nNOS, eNOS, and MAS1 in the A group are similar to those of the CY group. 

## 4. Discussion

### 4.1. Morphological Changes

In the current study, we used physiologically aged animals as a model for reproductive aging. In this disease-free model, the data revealed that supplementation with black chokeberry juice did not significantly affect the somato- and organo-metric parameters of rats. Although at the macroscopic level, there were no visible changes as a result of supplementation, the microscopic data convincingly indicated preservation of the tubule structure, reduction in the intertubular space, and the presence of Leydig cells surrounded by an abundance of blood vessels. Spermatogenic epithelium thickness in the A group showed a similar value to that observed in the CY group and CO group. However, the variation in the tubular area between the three experimental groups was very slight. The age-related changes in testicular histological structure that we found in the old versus young controls are similar to those described by other authors [5,46,47]. The morphological findings of the preserved seminiferous tubule structure in the supplemented animals correlated with the immunohistochemical findings. The higher intensity of neurotrophic factors in Leydig cells corresponds to their higher functional activity and corresponding levels of testosterone production. Testosterone is a potent stimulus for spermatogenesis. Another sign of increased functional activity of Leydig cells in the group of supplemented animals is the abundance of blood vessels seen adjacent to them. These gross anatomy data showed that changes in testicular weight normally occurred with age in rats, as described by other authors as well [5,46,48]. 

### 4.2. TRK-C and NT3 Immunoreaction 

Neurotrophin-induced functional activity of Leydig cells has already been well described in the literature [49]. Neurotrophins affect both somatic Sertoli and Leydig cells as well as the spermatogenic epithelium. Their modulating influences start during embryonic development and continue throughout the life of the male individual regulating reproduction [50]. Some studies indicate that the effects of certain neuronal growth factors, such as nerve growth factor (NGF) and neurotrophin-3 (NT3), may be critical for the development of adult Leydig cells [50]. NT3 binds to the TRK-C receptor, a member of the tyrosine protein kinase TRK family, and exhibits an anti-apoptotic effect [50]. Before birth, NT3 participates in regulating the formation of seminiferous cords and the differentiation of germ cells, as well as in determining the male sex. NT3 is secreted by Sertoli cells in the testes during embryonic development [51]. The NT3 promoter contains binding sites for the Sertoli cell transcription factor SOX9, and SOX9 stimulates the expression of NT3, which regulates the development of Leydig cells [52].

Our results showed a decreased intensity of TRK-C and NT3 receptor immunoreaction in the CO group compared to the CY group, which is an expression of age-related changes occurring in the testis. We found that under the influence of supplementation, the intensity of TRK-C and NT3 receptor immunoreaction increased, which could be an expression of stimulation and increased functional activity of Leydig cells. This could also lead to an amplification of testosterone secretion. The presence of a greater number of blood vessels in the vicinity of Leydig cells is another sign of their increased activity and also a sign of improved nutrition of the testis. 

Recently, it has been found that under conditions of oxidative stress due to aging, neurotrophins are oxidatively modified, thereby reducing their effectiveness [53]. On the other hand, there is evidence that polyphenols can enhance the synthesis of neurotrophic factors such as BDNF, NGF, neurotrophin 3 (NT3), and neurotrophin 4 (NT4), as well as increase their ability to bind directly to TRK-C receptors, regulating transcription, translation, proliferation, growth, and survival through pathways such as phosphatidylinositol 3-kinase (PI3K)/protein kinase B (AKT), mitogen-activated protein kinase (MAPK), signal transducer, and activator of transcription 3 (STAT3), activating cAMP response element-binding protein (CREB) [54]. There is a paucity of data in the literature on the effects of dietary polyphenols on neurotrophic factors in testes. Reports on the influence of functional foods on these factors have mainly focused on brain structure and function [55,56,57,58,59,60,61,62,63].

### 4.3. nNOS, eNOS, and Mas1 Immunoreaction

All three NOS isoforms are found in the testes, showing distinctive but overlapping patterns of cellular distribution. nNOS, iNOS, and eNOS are located in both Sertoli and germ cells in the seminiferous epithelium [64,65,66,67]. They are also found in Leydig cells [68], as well as in myoid cells, endothelial cells, myofibroblasts, and spermatozoa [64,65,67]. Since NOS is present in all types of cells in the testes, it appears to suggest that NO/NOS is necessary for spermatogenesis. Our results demonstrated the onset of an age-related decline in the intensity of the immunoreaction for nNOS and eNOS in Leydig cells and spermatogenic epithelium in the CO group compared with the CY group. In the A group, the intensity of the immunoreaction for nNOS and eNOS in Leydig cells and spermatogenic epithelium increased and approached that of young controls. This is a sign of activation of the NO synthesis process probably as a protective reaction in response to the increased oxidative stress occurring during aging. Until recently, it was not clear how NOS is regulated. Hormones and cytokines are the two classes of molecules known to regulate NOS in the testes. 

The expression levels of eNOS and iNOS vary under normal and pathological conditions. The overexpression of these two isoforms can induce destructive processes in reproductive tissues, such as low sperm motility and viability, activation of apoptosis in germ cells, and literally disrupting spermatogenesis. In both pathological and physiological processes, the paradoxical function of NO depends on the body’s overall state and on the oxidant/antioxidant balance mechanism [28].

The different RAS family members have been found in the human reproductive tract, including angiotensin II and angiotensin (1-7), as well as the expression of angiotensin II subtype-2 receptor (AT1R), angiotensin II subtype-2 receptor (AT2R), and proto-oncogene Mas receptors. All components are necessary to mediate the local effects of RAS on physiological and pathological processes, including maturation, fine-tuning of reproductive regulation, angiogenesis, and tumor cell proliferation [22,68,69].

ACE2 is the primary Ang-(1-7)-forming enzyme and can produce Ang-(1-7) from Ang II [70] or, less efficiently, by hydrolyzing Ang I to Ang-(1 –9) [71], with subsequent generation of Ang-(1–7) through ACE and neutral endopeptidase hydrolysis [72]. It has been established that Ang-(1-7) acts as an endogenous ligand for the G protein-coupled Mas receptor [73,74]. This cell surface receptor is highly expressed in the brain, heart, kidneys, endothelium, and testes [74]. Similar to the ACE2 expression pattern, Mas mRNA in the testes is found in Leydig and Sertoli cells, with much higher expression in Leydig cells [73]. Moreover, a recent study in Mas knockout mice found that Mas deletion affects the expression of enzymes involved in testosterone biosynthesis in Leydig cells (steroidogenic acute regulatory protein and 3β-hydroxysteroid dehydrogenase 1 and 6), suggesting a possible role of Mas in regulating androgen metabolism in the male reproductive system [74].

### 4.4. Antioxidants and Steroidogenic Function of Leydig Cells

Zhao et al. have recently demonstrated that oxidative stress and chronic inflammation are involved in the decline in testosterone production both in vivo and in vitro in aged Leydig cells. Their results highlight the importance of cyclooxygenase-2 inhibitors (COX2) in the regulation of the age-related decline in testosterone synthesis by providing evidence that the activation of two signaling pathways, nuclear factor kappa-light-chain-enhancer of activated B cells (NF-κB) and p38 mitogen-activated protein kinases (p38 MAPK), leads to COX2 upregulation and is functionally linked to the oxidative stress response and chronic inflammation commonly observed in aging [75]. The age-related accumulation of reactive oxygen species in testicles has been shown to be one of the main reasons for diminished semen quality [7,11,76].

Leydig cells are essential for steroidogenesis and spermatogenesis. They express cytochrome P450, the luteinizing hormone receptor (LH-R), and secrete androsterone [77]. The process of steroidogenesis is highly susceptible to ROS damage as this process generates ROS under basal conditions. In addition, ROS actions occur at the site where the steroidogenic process is modulated by cytochrome P450 enzymes [78]. On the other hand, Leydig cells, Sertoli cells, and testicular spermatogenic and somatic cells produce several immunoregulatory and proinflammatory cytokines in basal and inflammatory circumstances, such as interleukin-1 (IL-1), interleukin-6 (IL-6), and tumor necrosis factor (TNF) [79]. Their increased expression stimulates the production of ROS, especially H_2_O_2_, which leads to inflammation, exposing Leydig cells to oxidative stress [80]. Leydig cells have an intracellular antioxidant defense system that prevents cell damage by maintaining a balance between ROS and antioxidants [81]. However, when the Leydig cell repair mechanism is impaired due to extensive oxidative damage, the cells undergo programmed cell death, resulting in a reduced Leydig cell population and insufficient testosterone production [82].

ROS and proinflammatory cytokines are inextricably linked and maintain a cycle. ROS induce heat shock proteins, which stimulate proinflammatory cytokines and the expression of cell adhesion molecules (CAMs) [83]. These results lead to the stimulation of ROS-producing white blood cells (WBCs), such as leukocytes and resident cells (e.g., macrophages, endothelial cells, and fibroblasts) [84]. ROS accumulation, which is due to the overproduction of ROS or overconsumption of endogenous antioxidant enzymes, is mostly associated with damaged properties such as cellular dysfunction, DNA damage, mitochondrial damage, lipid peroxidation, and cell apoptosis [85].

Our study sheds light on an area that has been poorly investigated. Therefore, in the current manuscript, we only discuss the dietary polyphenols found in *A. melanocarpa* juice, namely anthocyanins, quercetin, catechins, chlorogenic acid, etc. As shown in Table 1, the juice was particularly rich in phenolic compounds with the cumulative content exceeding 11,000 mg/L. Hydroxycinnamic acids, represented by chlorogenic and neochlorogenic acids, were the predominant individual phenolic compounds, followed by anthocyanins (cyanidin-3-galactoside, cyanidin-3-glucoside, cyanidin-3-arabinoside, and cyanidin-3-xyloside). Plant flavonoids have been associated with numerous health benefits, including cancer prevention, reduced risk of cardiovascular and neurodegenerative diseases, and delayed symptoms associated with aging [40]. Having a chemical structure similar to cholesterol and other steroids, flavonoids can affect the production of androgens in Leydig cells. Therefore, since the early 1960s, more than 500 publications have reported the effects of various flavonoids on testosterone production. However, only recently, the molecular mechanisms of flavonoid effects on steroid synthesis have been partially elucidated [40]. Aging is associated with reduced steroidogenic acute regulatory protein (StAR) levels in adult Leydig cells, leading to incomplete mitochondrial cholesterol import and lower testosterone production. Age-related decline in testosterone production can be delayed by increasing StAR and/or cytochrome P450 family 11 subfamily A member 1 (Cyp11a1) gene expression via supplementation with flavonoids or their derivatives [86,87]. 

The results found by Hu et al. prove that all four types of anthocyanins studied can inhibit ROS generation, alleviate potential mitochondrial membrane damage, and contribute to increased testosterone production. Among them, Cy-3,5-diglu with di-glycoside performs best in terms of antioxidant capacity, ameliorates cellular dysfunction, and increases StAR expression [88].

Martin et al. discussed the enhancement of testicular steroidogenesis in detail through the use of flavonoids and isoflavonoids [34]. King et al. found a positive effect on steroidogenesis in Leydig cells of polyphenols such as quercetin, catechins, and anthocyanins, which are active constituents present in *A. melanocarpa* juice. CAMP-responsive element binding protein 1 (Creb1) is an important activator of the expression of steroidogenic genes including StAR in Leydig cells [89]. Cormier et al. reported that quercetin exhibited a positive effect on steroidogenesis by enhancing the transcriptional activity of Creb1 as well as the promoter activity of Cyp11a1 and gene—Ferredoxin 1 (Fdx1) [90]. Other authors have reported that quercetin increases StAR mRNA levels, StAR promoter activity, and steroid hormone production by MA-10 Leydig cells [91]. According to Wang, StAR expression and steroidogenesis in Leydig cells are also enhanced by blocking sex-determining region Y-box 2 (Sox2) signaling in response to quercetin [92].

One of the hallmarks of aging is an increased number of senescent cells secreting a variety of bioactive factors called senescence-associated secretory phenotype (SASP), and quercetin is among the first generation of senolytics targeting senescent cells [93]. Furthermore, Yu et al. reported that plasma testosterone levels increased 8 h after the administration of catechin, epicatechin, and epigallocatechin gallate in male rats [94]. As it is evident from the chemical composition data, black chokeberry juice is particularly rich in anthocyanins. Although these flavonoids have not been specifically studied in terms of their potential to regulate testosterone production, they could enhance steroidogenesis as it is known that they can inhibit COX2 activity and modulate MAPK pathway activity in Leydig cells, as both mechanisms affect StAR expression in Leydig cells [95,96]. 

## 5. Conclusions 

The data from our study demonstrate for the first time that supplementation with polyphenol-rich black chokeberry juice can slow down the aging process in rat testis. This intervention is observed to enhance the antioxidant potential of the testicular environment while concurrently promoting heightened functional activity in Leydig cells. The sustained integrity of the curved seminiferous tubules and increased blood vessel count, coupled with the heightened presence of neurotrophic factors and antioxidant molecules in the testicular milieu, serve as tangible evidence of *A. melanocarpa* juice’s anti-aging efficacy. *A. melanocarpa*, owing to its robust antioxidant properties, holds promise as a viable candidate for deployment as an anti-aging agent. By harnessing the potential of functional foods, the application of this fruit extends beyond the enhancement of testicular functionality to encompass broader health benefits. This holistic approach not only augments the well-being of the testes but also contributes to an overall enhancement of men’s health and quality of life. It should be noted that the study has some limitations, related to the fact that we did not investigate sperm parameters and apoptosis markers. However, this is an initial study that provides reliable information that can be the basis for further in-depth studies with more specific analyses. 

## Figures and Tables

**Table 1:** Polyphenol and sugar content and composition of *A. melanocarpa* fruit juice retrieved from [41].

**Phenolics**	**(mg/L)**
Total polyphenols (Folin–Ciocalteu)	11,237.4 ± 456.2
Quercetin	49.6 ± 3.2
Quercetin-3-β-glucoside	228.8 ± 11.0
Rutin	446.5 ± 12.5
Epicatechin	408.2 ± 25.6
Cyanidin-3-galactoside	1498.4 ± 102.3
Cyanidin-3-glucoside	120.1 ± 8.7
Cyanidin-3-arabinoside	501.9 ± 31.8
Cyanidin-3-xyloside	4.59 ± 0.2
Chlorogenic acid	1375.6 ± 80.3
Neochlorogenic acid	1543.1 ± 111.2
**Sugars**	**(g/L)**
Fructose	35.8 ± 2.1
Glucose	28.0 ± 2.7
Sorbitol	105.8 ± 7.0
Sucrose	1.1 ± 0.1

**Table 2:** Comparison of right testis weight.

Group	Right Testis	
	Absolute Weight, [g]	Relative Weight, [%]
CY	0.91 ± 0.31 ^a^	0.57
CO	1.64 ± 0.28 ^b^	0.41
A	1.72 ± 0.29 ^b^	0.41

Results are expressed as mean values ± standard deviation. There are no significant differences among values marked with the same superscript letters (*p* < 0.05). CY—young controls; CO—old controls; A—*A. melanocarpa* juice-supplemented group.

**Table 3:** Effect of *A. melanocarpa* supplementation on the count of germ cells.

Group	Number of			
	Seminiferous Tubules in Microscopic Field (100×)	Spermatogonia	Spermatocytes	Spermatids
CY	14.3 ± 0.23 ^b^	117.15 ± 5.21 ^b^	472.33 ± 32.14 ^b^	787.39 ± 18.11 ^b^
CO	10.5 ± 0.42 ^a^	93.62 ± 2.74 ^a^	316.21 ± 14.12 ^a^	554.27 ± 13.76 ^a^
A	13.1 ±0.39 ^b^	109.17 ± 3.73 ^b^	458.57 ± 25.15 ^b^	759.18 ± 21.7 ^b^

Results are expressed as mean values ± standard deviation. There are no significant differences among values marked with the same superscript letters in individual columns (*p* < 0.05). CY—young controls, CO—old controls, and A—*A. melanocarpa* juice-supplemented group.

**Table 4:** Immunoexpression intensity for NT3 receptor and TRK-C in rat testis. Semiquantitative analysis.

Group	Testis	NT3 Receptor Immunoexpression Intensity	TRK-C Immunoexpression Intensity
CY	Leydig cells	+++	+++
CO	Leydig cells	+	+
A	Leydig cells	++	++

Legend: - absent; + weak: ++ moderate; +++ strong expression. CY—young controls, CO—old controls, and A—*A. melanocarpa* juice-supplemented group.

**Table 5:** Immunoexpression intensity for nNOS, eNOS, and MAS1 in rat testis. Semiquantitative analysis.

	Testis	nNOS	eNOS	MAS1
CY	Germ cells	+++	+	++
	Leydig cells	+++	+	+
	Capillaries	+++	-	+++
CO	Germ cells	+	+	+
	Leydig cells	++	+	++
	Capillaries	++	-	+
A	Germ cells	++	+++	++
	Leydig cells	+++	++	++
	Capillaries	+++	-	+++

Legend: - absent; + weak: ++ moderate; +++ strong expression. CY—young controls, CO—old controls, and A—*A. melanocarpa* juice-supplemented group.

## Data Availability

Data are contained within the article.
